# Plasma *Plasmodium falciparum* Histidine-Rich Protein-2 Concentrations Are Associated with Malaria Severity and Mortality in Tanzanian Children

**DOI:** 10.1371/journal.pone.0035985

**Published:** 2012-05-07

**Authors:** Matthew P. Rubach, Jackson Mukemba, Salvatore Florence, Bernard John, Benjamin Crookston, Bert K. Lopansri, Tsin W. Yeo, Kim A. Piera, Stephen C. Alder, J. Brice Weinberg, Nicholas M. Anstey, Donald L. Granger, Esther D. Mwaikambo

**Affiliations:** 1 University of Utah School of Medicine and Veterans Affairs Medical Center, Salt Lake City, Utah, United States of America; 2 Hubert Kairuki Memorial University, Dar es Salaam, United Republic of Tanzania; 3 Loyola University Medical Center, Maywood, Illinois, United States of America; 4 Menzies School for Health Research and Charles Darwin University, Darwin, Australia; 5 Royal Darwin Hospital, Darwin, Australia; 6 VA Medical Centers and Duke University, Durham, North Carolina, United States of America; Biomedical Research Institute, United States of America

## Abstract

Plasma *Plasmodium falciparum* histidine-rich protein-2 (PfHRP-2) concentrations, a measure of parasite biomass, have been correlated with malaria severity in adults, but not yet in children. We measured plasma PfHRP-2 in Tanzanian children with uncomplicated (n = 61) and cerebral malaria (n = 45; 7 deaths). Median plasma PfHRP-2 concentrations were higher in cerebral malaria (1008 [IQR 342–2572] ng/mL) than in uncomplicated malaria (465 [IQR 36–1426] ng/mL; p = 0.017). In cerebral malaria, natural log plasma PfHRP-2 was associated with coma depth (r = −0.42; p = 0.006) and mortality (OR: 3.0 [95% CI 1.03–8.76]; p = 0.04). In this relatively small cohort study in a mesoendemic transmission area of Africa, plasma PfHRP-2 was associated with pediatric malaria severity and mortality. Further studies among children in areas of Africa with higher malaria transmission and among children with different clinical manifestations of severe malaria will help determine the wider utility of quantitative PfHRP-2 as a measure of parasite biomass and prognosis in sub-Saharan Africa.

## Introduction

Sequestration of parasitized red blood cells within the deep organ microvasculature is thought to explain the pathogenicity of *Plasmodium falciparum*, and results from the ability of parasitized erythrocytes to cytoadhere to endothelial cells in post-capillary venules [1]. Autopsies of cerebral malaria have demonstrated 26–40 times the burden of *Plasmodium falciparum* parasites in the deep tissue circulation of the brain compared to peripheral blood [2], [3]. This phenomenon is thought to explain the poor association between malaria severity and parasitemia measured by peripheral blood microscopy [4].

Histidine-rich protein-2 (PfHRP-2) is a protein synthesized by *P. falciparum* that facilitates heme polymerization to hemozoin [5]. The parasite releases PfHRP-2 as a water-soluble protein into the bloodstream of infected humans [5]. Quantitative PfHRP-2 has been identified as a metric of the total parasite biomass, as it includes antigen release from the sequestered parasite biomass, which is not accurately discernible on peripheral blood smear [4]. Plasma PfHRP-2 levels at presentation correlated with severity of malaria illness in adults from Thailand [4] and Indonesian Papua [6]. In both these series, plasma PfHRP-2 was an independent predictor of mortality [4], [6]. However, this has not yet been shown in African children. A recent study in Papua New Guinean children found no association between plasma PfHRP-2 and severity of malaria [7]. Some have proposed that differences in age, PfHRP-2 strain variation, and/or intensity of transmission may influence the utility of plasma PfHRP-2 in predicting parasite biomass. In Africa, where transmission is generally higher than Asia, persistent circulating plasma PfHRP-2 from recent infection has been hypothesized to potentially overestimate parasite biomass [7], given that blood PfHRP-2 can persist up to four weeks after successful drug treatment [8]. With high rates of asymptomatic parasitemia in Africa and Papua New Guinea, low plasma PfHRP-2 levels in children clinically diagnosed with severe malaria may suggest an alternative non-malarial cause of severe disease with incidental parasitemia.

Although there is a clear association with disease severity in Asia-Pacific adults, the greatest burden of severe malaria morbidity and mortality is in African children [9]. To examine the association between malaria severity and parasite biomass in this population, we measured plasma PfHRP-2 levels in children with cerebral malaria and uncomplicated malaria in a mesoendemic transmission area of Tanzania [10]. We hypothesized that plasma PfHRP-2 would be associated with disease severity and death.

## Methods

### Study Design and Site

We conducted a prospective observational study in Dar Es Salaam, Tanzania, a city (population ∼3.2 million) with mesoendemic transmission of falciparum malaria [10]. Children with uncomplicated or cerebral malaria were enrolled from the clinics and the inpatient wards of Amana and Mwanayamala Districts Hospitals and commenced antimalarial therapy. The distance between these two district hospitals is 6.5 kilometers, and both are located in central areas of Dar es Salaam. Once enrolled, children with cerebral malaria were transferred to the clinical research unit at the Hubert Kairuki Memorial University Hospital for further evaluation and care. The sample sizes in both the uncomplicated and cerebral malaria groups were comparable to those previously found sufficient by Yeo et al to demonstrate a significant relationship between plasma PfHRP-2 and both disease severity and mortality in adults [6]. Approval for this study was obtained from the institutional review boards of Hubert Kairuki Memorial University, National Medical Research Institute, Tanzania, University of Utah, Loyola University, and Duke University. Informed consent was obtained from parents or guardians of all children; human experimentation guidelines of the U.S. Department of Health and Human Services were followed.

### Patients and Sampling

Children were aged from 6 months to 6 years. Inclusion criteria for cerebral malaria were defined by WHO: any level of *P. falciparum* parasitemia on peripheral blood smear; unarousable coma (Blantyre Coma Score ≤2) not attributable to hypoglycemia (blood glucose level <40 mg/dL) and persisting more than 60 minutes after any convulsion; no other identifiable cause of coma [1]. Inclusion criteria for those with uncomplicated malaria were: a clinical syndrome consistent with malaria and a documented fever (temperature ≥38°C) or history of fever within 48 hours from time of enrollment; *P. falciparum* parasitemia >10,000 parasites/µL on Giemsa-stained blood film and positive *P. falciparum* rapid diagnostic test (Paracheck-Pf [Omega Diagnostics]); and no other cause of fever identified.

Exclusion criteria for both groups with malaria were any of the following: microscopic evidence of mixed infection with any other *Plasmodium* species; bacterial co-infection as evidenced by a positive blood, urine or cerebrospinal fluid (CSF) culture; CSF analysis indicative of bacterial meningitis; quinine or artemesinin combination therapy initiated >18 hours prior to enrollment; and/or hemoglobin <5 mg/dL. Erythrocyte transfusions were not readily available at our study sites. Children were also excluded from enrollment as uncomplicated malaria if they exhibited WHO warning signs suggestive of severe disease: inability to suckle, eat and/or drink; excessive vomiting; abnormal respiratory rate or respiratory distress as evidenced by accessory muscle use, suprasternal retractions, or intercostal retractions; recent history of convulsions; altered mental status; or inability to sit unaided.

### Clinical Evaluation and Management

At presentation, demographic information, clinical history, and examination were documented using standardized case report forms. Capillary blood samples were obtained for Giemsa-stained thick and thin blood smears as well as on-site rapid diagnostic testing (Paracheck-Pf [Omega Diagnostics]). Venous samples for routine laboratory analysis included complete blood count (Beckman-Coulter, Model Act 10), sodium, potassium, chloride, bicarbonate, blood urea nitrogen, creatinine, and venous blood gas (i-STAT® 1 [Abbott Laboratories]). Blood cultures were obtained in all children with uncomplicated and cerebral malaria to rule out concomitant bacteremia. Lumbar puncture with opening pressure was performed in all patients with coma to evaluate for meningitis. Cerebrospinal fluid analysis included the following: glucose and protein levels; cell count with differential by trained microscopists; and bacterial culture.

Plasma PfHRP-2 was measured by ELISA as previously described [6] using primary and secondary monoclonal antibodies to *P. falciparum* HRP-2 (MPFM-55A and MPFG-55P, Immunology Consultant Laboratories). Concentrations were derived from standard curves utilizing purified PfHRP-2 kindly provided by D Sullivan (Johns Hopkins School of Public Health). Samples with ODs outside the linear part of the curve were repeated at an appropriate dilution until an accurate concentration was determined. The lower limit of detection was 1.5 ng/mL.

Children with uncomplicated malaria and cerebral malaria received anti-malarial therapy and other supportive care as per standard Tanzanian national protocols (artemesinin combined therapy and IV quinine, respectively). Treatment was initiated as soon as the diagnosis of malaria was suspected. Children with cerebral malaria were re-examined daily until death or hospital discharge.

### Statistical Methods

Statistical analysis was performed using SAS (version 9.2). Results are presented as mean with 95% confidence intervals for normally distributed continuous variables or median with interquartile range for variables with non-parametric distribution. Non-parametric tests and natural log transformation of variables were used when the assumption of normality was violated. Differences between PfHRP-2 values in uncomplicated malaria and cerebral malaria cases were compared using Wilcoxon rank-sum scores two-sample test. Further comparisons of PfHRP-2 values between uncomplicated malaria, cerebral malaria survivors, and cerebral malaria fatal cases were performed using a Kruskal-Wallis test as well as the Wilcoxon rank-sum tests with the Holm’s Step Down Procedure for partitioning alpha. Differences between normally distributed continuous variables were compared using Student’s t-test. Correlation between variables was evaluated using Spearman’s correlation coefficients. In the cerebral malaria group, logistic regression was used to determine the association between death and plasma PfHRP-2 concentrations and clinical variables previously shown to be associated with mortality (coma score and plasma bicarbonate) [11]. Goodness-of-fit was assessed by the Hosmer-Lemeshow test.

## Results

We screened 227 febrile children for uncomplicated malaria between November 2007 and January 2011. Of these, 67 were consented, six were excluded because they did not meet inclusion/exclusion criteria, leaving 61 enrolled participants (49 and 12 from Amana and Mwanayamala District Hospitals, respectively). We screened 153 febrile children with central nervous system conditions (mostly stupor or coma) for cerebral malaria. Fifty-one met inclusion criteria for cerebral malaria and were consented. Of these, six were excluded, leaving 45 enrollees (23 and 22 from Amana and Mwanayamala District Hospitals, respectively). Baseline characteristics comparing the two groups are shown in Table 1. There were no significant differences between the two groups in age, sex, weight, parasitemia on blood smear, blood glucose, or plasma bicarbonate. Children with cerebral malaria had a significantly higher respiratory rate and plasma creatinine and a longer duration of illness at the time of enrollment. Children with cerebral malaria also had significantly lower axillary temperature and hemoglobin concentration. As per exclusion criteria, no children had concomitant bacteremia or meningitis.

**Table 1:** Baseline characteristics of children with uncomplicated malaria and cerebral malaria.

Characteristic	Uncomplicated Malaria (n = 61)	Cerebral Malaria (n = 45)	P-value
Age, months	44 (38–49)	51 (46–56)	0.06
Male sex, no. (%)	30 (53.6)	15 (34.9)	0.07
Illness Duration at Enrollment, days	2.42 (1.98–2.86)	3.14 (2.64–3.64)	0.03
Weight, kg	14.2 (13–15.2)	15.4 (14.2–16.5)	0.12
Axillary temperature, °C	39.1 (38.8–39.3)	38.3 (38–38.6)	<0.001
Respiratory rate	37 (35–40)	42 (38–46)	0.03
*P. falciparum* peripheral parasitemia,parasite/µL; median (inter-quartile range)	94,451 (32,440–211,265)	41,782 (7126–128,230)	0.06
Hemoglobin, g/dL	8.4 (7.8–9.1)	7.1 (6.6–7.7)	0.004
Blood glucose, mg/dL	119 (96–143)	120 (106–135)	0.92
Plasma bicarbonate, mmol/L	19.4 (16–22.7)	20.5 (19.3–21.7)	0.46
Plasma creatinine, mg/dL	0.3 (0.3–0.4)	0.7 (0.5–0.8)	0.001

**Figure 1:**
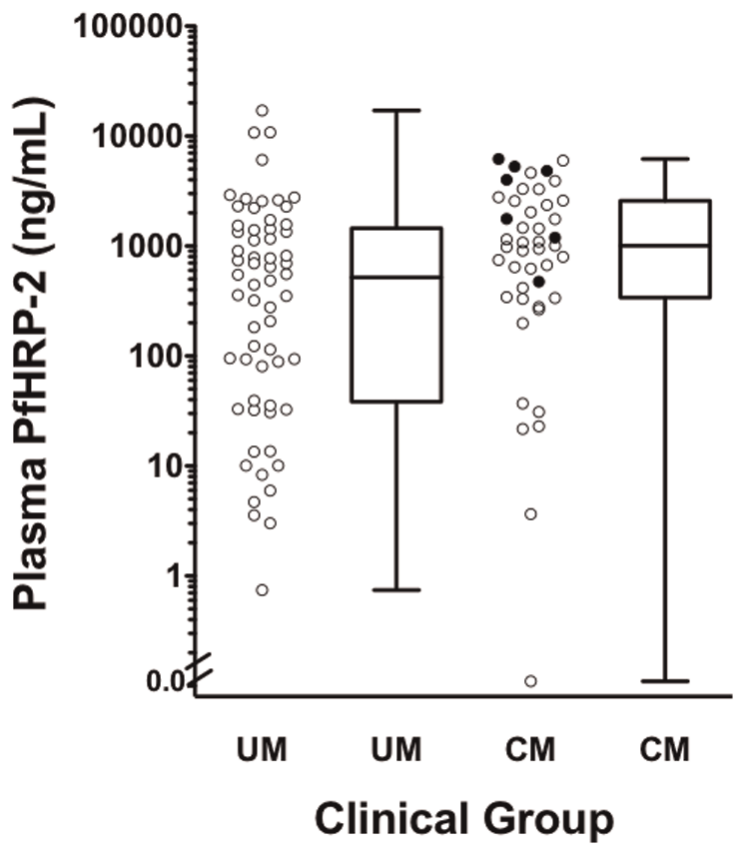
Plasma *P. falciparum* histidine-rich protein-2 concentration in children with uncomplicated and cerebral malaria. Dot plots showing plasma PfHRP-2 concentration value; box plots showing median, interquartile range, maximum and minimum for plasma PfHRP-2 in uncomplicated malaria (UM) and cerebral malaria (CM). Open circles  =  recovery, closed circles  =  death.

The median concentration of plasma PfHRP-2 in cerebral malaria was significantly higher than in uncomplicated malaria (1008 ng/mL [IQR 342–2572 ng/mL] and 443 ng/mL [IQR 36–1426 ng/mL], respectively, p = 0.02) (Figure 1). One patient in each group had undetectable levels of PfHRP-2. In those with cerebral malaria, six patients (13%) were outliers, having PfHRP-2 concentrations at least an order of magnitude lower than the rest of the cohort (Figure 1). There was no significant difference in mean age of the six cerebral malaria outliers (55 months [95% CI 44.1–66.9]) with the mean age in the rest of the cerebral malaria cohort (50 months [95% CI 44.6–55.4]; p = 0.49).

**Figure 2:**
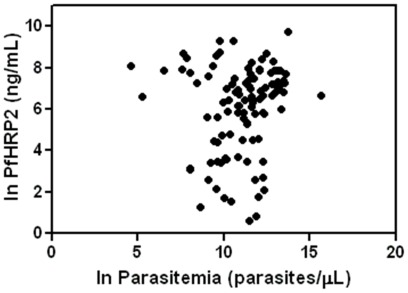
Lack of correlation between plasma PfHRP-2 and peripheral blood parasitemia in children with uncomplicated and cerebral malaria.

On univariate analysis, natural log plasma PfHRP-2 concentration correlated with lower Blantyre Coma Score (r = −0.44; p = 0.003) and with hemoglobin concentration (r = −0.47; p<0.0001), but not with plasma creatinine (r = 0.09, p = 0.54). Natural log plasma PfHRP-2 concentration correlated with the natural log peripheral parasitemia in uncomplicated malaria (r = 0.41, p = 0.001), but did not correlate with natural log peripheral parasitemia in cerebral malaria (r = −0.09, p = 0.54) or in an aggregate analysis of all patients enrolled (r = 0.16, p = 0.11) (Figure 2).

Seven children died, all of whom had cerebral malaria. Median plasma PfHRP-2 concentrations in those with fatal outcomes (4000 ng/mL [IQR 1194.5–5287.3 ng/mL]) were higher than in those who survived (926.1 ng/mL [IQR 330.7–2028.3]) (Wilcoxon rank-sum, Holm’s Step Down Procedure, p = 0.034). A comparison of plasma PfHRP-2 values between uncomplicated malaria cases, cerebral malaria survivors, cerebral malaria fatal cases found a significant difference among the three groups (Kruskal-Wallis, p = 0.02). In patients with cerebral malaria, increased natural log plasma PfHRP-2 was associated with an increased risk of death on univariate analysis (OR 3.0 [95% CI 1.03–8.76]; p = 0.04). Coma score and plasma bicarbonate were not significantly associated with death. In multivariate models natural log PfHRP-2 remained significantly associated with death (p = 0.04) while natural log peripheral parasitemia and plasma bicarbonate did not (p = 0.13 and p = 0.18, respectively).

## Discussion

Plasma concentration of PfHRP-2, a measure of total parasite biomass, was associated with malaria severity, depth of coma and mortality in Tanzanian children living in an area with mesoendemic malaria transmission. This supports similar findings in adults from areas of relatively unstable transmission in Asia and Indonesian Papua [4], [6]; however it is particularly significant because it extends the association between parasite biomass and disease severity to African children, the demographic sub-group with the greatest burden of severe malaria morbidity and mortality [9]. PfHRP-2 levels did not correlate with parasitemia based on peripheral blood microscopy in children with cerebral malaria. These findings support the assertion that PfHRP-2 is more accurate than peripheral blood smear in measuring the total body parasite biomass in both children and adults. While we found a significant difference in median values between cerebral malaria and uncomplicated malaria, and an association with depth of coma and mortality in cerebral malaria, quantitative plasma PfHRP-2 did not clinically discriminate between cerebral malaria and uncomplicated malaria, as the range of values in the two groups overlapped. Nonetheless, in a population of subjects with malaria, quantitative PfHRP-2 gives clinical researchers a measure that controls for and/or compares parasite biomass in pathophysiologic studies of biomarkers or mediators of severe malaria. In addition to this utility in clinical research, our finding that PfHRP-2 was predictive of mortality suggests that quantitative PfHRP-2 may have prognostic value in children presenting with cerebral malaria.

Two previous studies have measured plasma PfHRP-2 in children with severe malaria. A small Kenyan study did not relate PfHRP-2 with disease severity or outcome [12]. A recent study from Papua New Guinea found no correlation between PfHRP-2 and severity of malaria infection among children with severe malaria in a relatively high transmission area [7]. The reasons for the lack of association in this study are not clear. The Papua New Guinea study had an unusually low case fatality rate among patients enrolled with severe malaria (<0.5%), suggesting less severe disease, and used recombinant PfHRP-2 was used for ELISA quantitation rather than the purified PfHRP-2 used in all three studies showing associations with disease severity and mortality [current study,4, 6]. In contrast to the Papua New Guinea study, we (and the adult studies) found no association between plasma PfHRP-2 and creatinine to suggest slower renal clearance in children. Geographic variation in parasite PfHRP-2 polymorphisms may contribute [13], although plasma PfHRP-2 has been associated with disease severity and mortality elsewhere on the island of New Guinea [6]. It is also possible that differences in comorbidities or malaria transmission may contribute. With relatively high rates of asymptomatic parasitemia in coastal Papua New Guinea, low plasma PfHRP-2 levels in children clinically diagnosed with severe malaria could occur with non-malarial causes of severe illness associated with incidental parasitemia. Nevertheless, we found that plasma PfHRP-2 is associated with pediatric malaria severity and mortality in a mesoendemic transmission area of Africa.

Strengths of our study include rigorous exclusion of bacteremia and meningitis that may mimic severe malaria. A limitation lies in the reliability of the WHO clinical case definition for cerebral malaria. An autopsy study (without blood culture exclusion of bacteremia) found that the clinical definition has a specificity of only 77% [14]. Among children in our study with clinically defined cerebral malaria and exclusion of bacteremia, there were six low outliers (13%) who had plasma PfHRP-2 levels at least an order of magnitude below the rest of the cerebral malaria group. We speculate that the low PfHRP-2 values in these children could reflect coma from another source with coincidental *P. falciparum* parasitemia, higher levels of host neutralizing antibody to PfHRP-2, or a dysregulated host inflammatory response despite low burden of infection. Another limitation of our study is the relatively small sample size, which could lead to a type I error (i.e. rejecting the null hypothesis when in fact the null hypothesis is true).

We conclude that plasma PfHRP-2 is associated with malaria severity and mortality in Tanzanian children from a mesoendemic area of transmission. Although further studies are needed in other areas of Africa with higher malaria transmission and in children with different clinical manifestations of severe malaria, our findings support the utility of plasma PfHRP-2 in clinical research to estimate the total parasite biomass in falciparum malaria, in children as well as adults.
